# The Close Interconnection between Mitochondrial Dynamics and Mitophagy in Cancer

**DOI:** 10.3389/fonc.2017.00081

**Published:** 2017-05-02

**Authors:** Matteo Bordi, Francesca Nazio, Silvia Campello

**Affiliations:** ^1^Department of Biology, University of Rome Tor Vergata, Rome, Italy; ^2^Department of Pediatric Hematology and Oncology, IRCCS Bambino Gesù Children’s Hospital, Rome, Italy; ^3^IRCCS, Fondazione Santa Lucia, Rome, Italy

**Keywords:** cancer, mitochondrial dynamics, autophagy, mitophagy, mitochondrial fate

## Abstract

Recent decades have revealed the shape changes of mitochondria and their regulators to be main players in a plethora of physiological cell processes. Mitochondria are extremely dynamic organelles whose highly controlled morphological changes respond to specific and diverse pathophysiological needs. Thus, their qualitative control is crucial for the determination of cell function and fate. Moreover, ever-new metabolic changes, mainly attributable to mitochondrial (dys)functions, are strongly connected to cancer and its microenvironment. For this reason, the aspects controlling mitochondria activity and status are in the oncological spotlight. In this review, we elucidate the most intriguing discoveries related to two apparently independent but strictly interconnected processes crucial for the organelle functionality and fate, mitochondrial dynamics, and mitophagy. We will mostly focus on their metabolic interconnections and regulations that can causally foster a tumoral context.

## The Mitochondria Ballet

A large body of evidence has highlighted the existence of a close interconnection between the cancer cells fate and the two apparently unrelated cellular processes of mitochondrial dynamics and autophagy. Indeed, cancer cells regulate the morphology of their mitochondria on the basis of their bioenergetic and biosynthetic needs to sustain proliferation and migration, and to escape apoptosis. The modulation of the organelles shape is also crucial for the qualitative control of the same, which mainly depends on the selective autophagic process called mitophagy.

**Figure 1:**
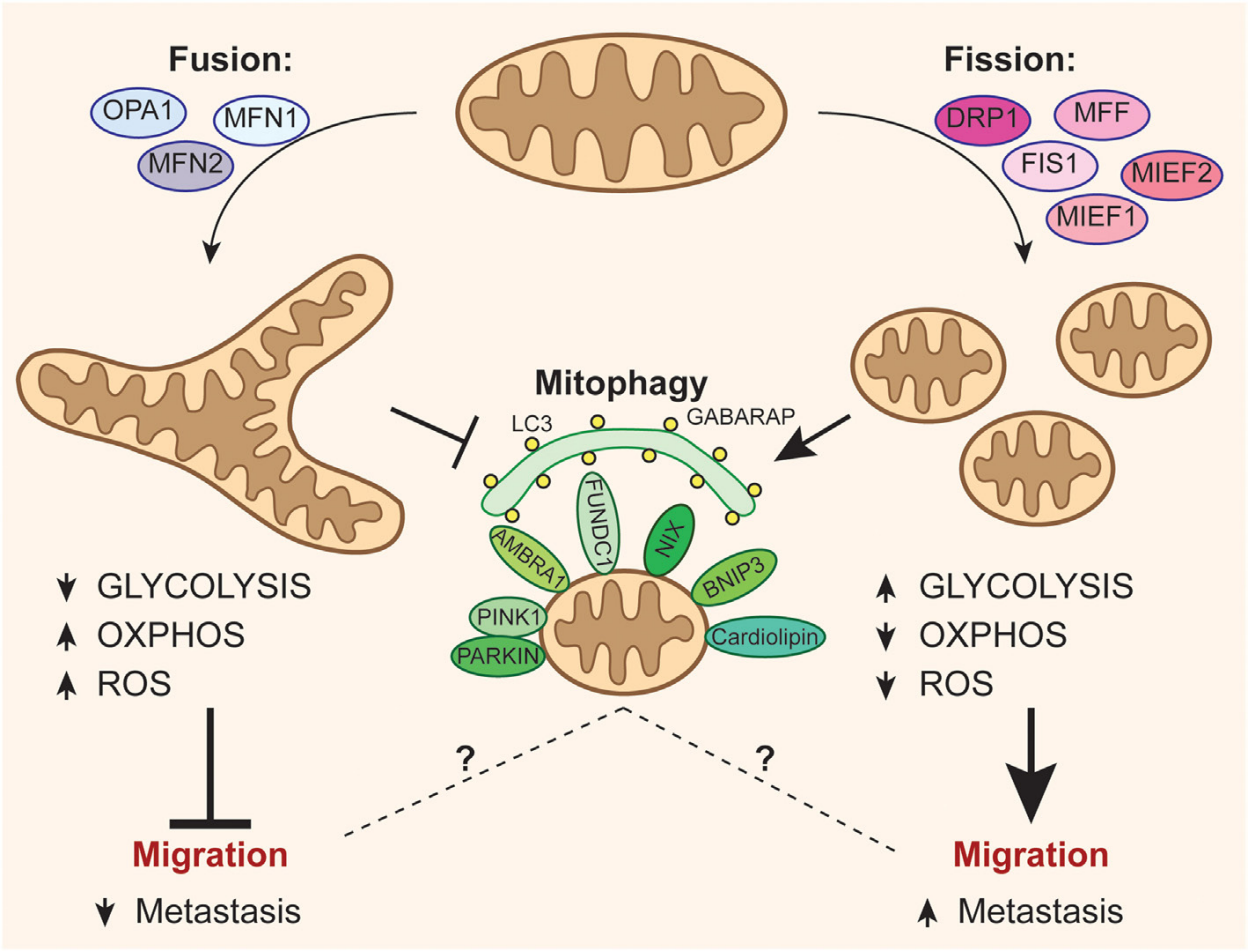
**Mitochondrial dynamics and fate can affect metastatization of cancer cells**. The activation of optic atrophy 1–mitofusin (MFN)1/2-mediated mitochondrial fusion promotes oxidative phosphorylation (OXPHOS) at the expense of anaerobic glycolysis, leading to increased reactive oxygen species (ROS) levels and, consequently, disfavoring the migration of cancer cells. On the other hand, mitochondrial fission events mediated by dynamin-related protein-1 (DRP1) recruitment through its accessory proteins (mitochondrial fission factor, FIS1, MIEF1/2) inhibit oxidative metabolism by increasing the energetic yield of glycolysis, and reduces ROS production, so easing the process of metastasis. Moreover, fission is critical for the correct activation of mitophagy, even though it remains ambiguous how mitophagy may be related to tumor migration.

Mitochondria are highly dynamic organelles, forming an active network capable of undergoing sudden changes to adapt its structure to the energetic and physiological needs of the cell (1). Mitochondria shape results from a balance between fusion and fission mechanisms in response to endogenous and exogenous stimuli or insults (2). These organelles are fundamental for: (i) energy production through an efficient oxidative phosphorylation (OXPHOS) and ATP production and (ii) biosynthetic metabolism and production of metabolites (3). On the other hand, mitochondria are the major source of reactive oxygen species (ROS) that could cause oxidative damage to proteins, lipids, and DNA (4) and result in aging and several diseases, including neurodegenerative disease, diabetes, and cancer (5, 6) see Figure 1.

### Mitochondrial Fusion Dynamism

In the fusion phase, the outer and inner mitochondrial membranes (OMM and IMM, respectively) of two distinct mitochondria fuse with each other, so resulting in the mixing of the mitochondrial content (7). Fusion is regulated by the activity of dynamin-related GTPases Mitofusin (MFN) 1 and 2, and by optic atrophy 1 (OPA1) (1). Upon GTP binding, the GTPase domains of MFN1 and MFN2 interact, forming homo- and hetero-dimers that undergo conformational changes upon GTP hydrolysis, bringing the opposing OMM into close contact to foster their fusion (8–11). OPA1, instead, facing the inner membrane space, regulates the IMM fusion (1), together with MFN1 (12). The pleiotropic OPA1 is also involved in mitochondrial *cristae* architecture, mitochondrial bioenergetics, and apoptosis (13–15). Distinctive OPA1 isoforms have been identified, derived by differential RNA splicing and precursor protein processing (16) [for more details, see Kasahara and Scorrano (1)]. Moreover, OPA1 activity is regulated by a proteolytic cascade; the long isoform, i.e., characterized by pro-fusion activity, can be cleaved by: the large intermembrane space AAA-protease Yme1L (17, 18), the mitochondrial matrix ATPase proteases (19), the metalloprotease paraplegin (20), and OMA1 (19, 21). The shorter isoform is a substrate for the presenilin-associated rhomboid-like (PARL) and contributes to regulate the *cristae* morphology and apoptosis (1). It is possible that tissue-specific expression of OPA1-cleaving proteases and/or splice variants contributes to the complex pattern of OPA1 processing. Fusion processes are inhibited upon specific stress stimuli (such as mitochondrial uncoupling agents like CCCP), promoting the activation of the opposite fission pathway; when mitochondria lose membrane potential [ΔΨ(m)] or the ATP levels decrease, OMA1 is stabilized and enhances the cleavage of OPA1 so preventing inner membrane fusion (21). As the long and short OPA1 isoforms are both required for fusion, complete conversion of OPA1 to the short isoform by OMA1 shuts off the fusion machinery of dysfunctional mitochondria. This activity could contribute to a quality control process by preventing fusion of defective mitochondria. Under the same circumstances, fusion can be prevented by MFNs degradation mediated by both PTEN-induced putative kinase 1 (PINK1) and PARKIN during mitophagy, a selective removal of damaged mitochondria by macroautophagy (described in detail in the next section) (22). Likewise, in response to genotoxic stresses, MFN2 is phosphorylated by Jun N-terminal kinase (JNK), ubiquitinated by the E3 ubiquitin ligase HUWE1 and consequently degraded by the proteasome, leading to mitochondrial fragmentation and apoptotic cell death (23).

### Mitochondrial Fission Dynamism

As mentioned above, when fusion is inhibited, fission is initiated. Fission is mediated by the activation of dynamin-related protein-1 (DRP1), a protein mainly localized in the cytosol that cycles between the cytosol and the OMM, where it constricts and cuts the organelles (16).

Many pathways regulating the activities of the fission machinery in mammalian cells have been discovered. Also DRP1 activity is reversibly regulated by several post-translational modifications, in particular by phosphorylation/dephosphorylation events. The phosphatase calcineurin dephosphorylates DRP1 on Ser637 (24) leading to its translocation to OMM, where it interacts with different accessory factors such as the mitochondrial fission factor (MFF), FIS1, Mid49/Mief1, and Mid51/Mief2 that may act in various ways to promote DRP1-dependent fission (16, 25). Once on the OMM, DRP1 assembles into multimeric spiralic structures and, through hydrolysis of GTP, mediates constriction of the spiral and scission in two distinct mitochondria (16, 26). Three protein kinases that phosphorylate different DRP1 serine residues have been identified: (1) high-glucose-induced Ca^2+^, extracellular signal-regulated kinase 1/2 (ERK1/2) causing mitochondrial fission (27); (2) the protein kinase A (PKA) that inhibits DRP1 GTPase activity and therefore prevents fission (28); and (3) a kinase anchoring protein 1 (AKAP1), which upon hypoxia conditions stabilizes PKA on neurons OMM, so exacerbating PKA negative regulation of DRP1 and promoting both mitochondrial elongation and neuronal survival (29). Opposed to PKA/AKAP1, PP2A/Bβ2-mediated dephosphorylation of Drp1 enhances fission, so regulating neuronal development *via* mitochondrial bioenergetics (30). During mitosis, CDK1/cyclin B leads to mitochondrial fragmentation activating DRP1 *via* its phosphorylation at Ser616, thereby facilitating an appropriate distribution of mitochondria to daughter cells (31). Furthermore, it has been described that the E3 ligase PARKIN (32) and another E3 ligase, MARCH-V (33), mediate the degradation of DRP1 *via* proteasome, while positively modulating MFN2, probably thus boosting fusion. Mitochondrial-anchored protein ligase is a mitochondrion-anchored small ubiquitin-like modifier (SUMO) ligase that sumoylates DRP1 to stimulate mitochondrial fission (34). Removal of SUMO from DRP1 involves sentrin-specific protease 5 (SENP5), a SUMO protease that recognizes several mitochondrial targets (35). Despite many pieces of the mitochondrial dynamics machinery puzzle being now in place, further pieces will probably be discovered and need to be added to better explain the role of all the components regulating the fusion and fission pathways.

### Mitochondrial Dynamics-Related Modulation of Mitochondria Quality versus Efficiency

The mitochondrial shape remodeling is intimately connected to the fate of the organelles; in fact, the mitochondrial function is maintained by the coordinated activation of the mitochondrial quality control pathway that guarantees the degradation of damaged mitochondria, so safeguarding the cell from the activation of apoptosis (1). The selective removal of damaged mitochondria is ensured by macroautophagy (hereafter referred to as autophagy): it is an evolutionarily conserved process, which triggers degradation of bulk cytoplasm, long-lived proteins, and entire organelles *via* lysosome, for recycling purposes (36, 37). Autophagy is upregulated during cellular stress (38) (increase of oxidative stress, ER-stress, DNA damage) and is closely linked to the mechanisms underlying aging (39). Autophagy is a very dynamic process described as a flux (40). The “autophagy core complex,” a multimolecular machinery including the ULK1 complex and the BECN1-VPS34 complex, initiates the formation of a double-membrane vesicle, termed autophagosome (AP), which surrounds the substrates (37); the matured AP is thus delivered to lysosomes for degradation (41). Several kinase complexes, including MTOR complex 1 that negatively affects ULK1 activity (42), tightly regulate each step of autophagy (36). Whereas the autophagy response to starvation is bulk degradation of cytosolic material, other types of stress, such as damaged organelles or aggregated proteins (39), require selective sequestration of the specific cargo into the autophagosomal membranes. Selectivity is achieved through autophagy receptors, such as SQSTM1/p62, NBR1, NDP52, OPTINEURIN (OPTN), TAX1BP1, and NIX, which recognize, on the one hand, cargos tagged by degradation signals and, on the other hand, the autophagosomal membrane through their LC3-interacting regions (LIR) (43, 44). The specific autophagic breakdown of mitochondria is termed mitophagy (45). In this case, mitochondria with decreased membrane potential are less likely to be engaged in subsequent fusion events and, instead, are prone to be removed through mitophagy. By contrast, inhibition of fission impedes mitophagy and results in decline of the respiratory capacity, whereas, arrest of autophagy leads to the accumulation of mitochondria with low membrane potential and low OPA1 (46). Thus, as already mentioned, mitophagy and mitochondrial dynamics are tightly interconnected. In recent years, distinct mitophagic pathways have been identified, revealing that the elimination of altered mitochondria is a critical step for mammalian cell fate.

### Spotlight on the Molecular Pathways

PTEN-induced putative kinase 1 (PINK1)–PARKIN-dependent mitophagy is the most characterized mitophagic pathway. In basal healthy conditions, PINK1 is constitutively cleaved by the mitochondrial processing protease (MPP) and, further, cleaved by PARL and so rapidly degraded (47, 48). Upon mitochondria membrane depolarization, MPP and PARL are inhibited and PINK1 is consequently stabilized at the OMM (49, 50); this leads to its autophosphorylation (50, 51) and to the phosphorylation of its substrates that initiates mitophagy. In particular, PINK1 phosphorylates ubiquitin at its Ser65; this leads to the activation and recruitment to mitochondria of the E3 ligase PARKIN (52–54), thus amplifying the signal started by PINK1 (55). Fascinatingly, a recent study shows that, in HeLa cells, PINK1 phosphorylates and also recruits NDP52 and OPTINEURIN, two autophagic cargo receptors that, despite being considered downstream of AP formation, are important for re-localization of ULK1 on damaged mitochondria (55). Moreover, as previously mentioned, PINK1 mediates the phosphorylation of MFN2 that acts as a relevant receptor for a correct PARKIN translocation (56).

Following its translocation to the mitochondrial surface, PARKIN modifies many OMM proteins by both K48- and K63-linked ubiquitin chains (57–59). On the one hand, PARKIN promotes proteasome-degradation of MFN1, MFN2, some TOM complex proteins (TOM20) (22), and mitochondrial rhoGTPase-1 (MIRO1) (60), so favoring the mitochondrial fission and arresting mitochondria motility; on the other hand, PARKIN mediates K63-linked ubiquitination of substrates such as VDAC1, a mitochondrial protein voltage-dependent anion channel, which acts as a signal for recruiting autophagic cargo receptors (22, 61, 62). A very recent publication has opened new insights into the mitophagy mechanisms, revealing that an IMM protein prohibitin 2 (PHB2) acts as a mitophagy receptor directly binding to LC3 through its LIR motif and is essential for PARKIN-mediated mitophagy (63).

Recruitment of PARKIN to depolarized mitochondrial membranes is inhibited by the anti-apoptotic proteins BCL-XL, Mcl-1, and BCL2, in a BECLIN1-independent manner (64, 65). Additionally, the contribution of deubiquitinating enzymes (DUBs) on the PINK1/PARKIN-mediated mitophagy has been characterized. DUBs regulate ubiquitin signals by removing or trimming ubiquitin linkages, and thus they can play important roles in modulating mitophagy. The OMM-localized DUB ubiquitin-specific processing proteases USP30 (66) and USP15 (67) antagonize PARKIN activity by cleaving ubiquitin chains on mitochondria (22). USP8, instead, antagonizes mitophagy by directly deubiquitinating PARKIN, so contributing to its recruitment and activity (68). Moreover, Heo et al. report that mitochondrial damage activates the kinase TBK1, which phosphorylates autophagy adaptors OPTN, NDP52, and SQSTM1/p62, so enhancing their recruitment to damaged mitochondria to promote mitophagy (44). Collectively, these events constitute a feedforward amplification mechanism to endorse mitophagy (44, 58).

Although PINK1–PARKIN-dependent mitophagy is the most characterized and most tumors-linked pathway, as mentioned, alternative pathways have been recently described, and different cell types might have different degrees of sensitivity for the activation of characteristic mitophagic pathways. For instance, PARKIN is further reported to interact with Activating molecule in BECN1-regulated autophagy protein 1 (AMBRA1) that activates BECLIN1 complex, endorsing the AP formation nearby (69). However, AMBRA1 can also induce mitophagy regardless of PARKIN and PINK1, *via* a direct interaction with LC3 (70) and allegedly, *via* its positive loop on ULK1 (42). In fact, a recent study points out that, upon mitophagy induction by either hypoxia or mitochondrial uncouplers, ULK1 translocates to mitochondria and phosphorylates the cargo receptor FUN14 domain-containing protein, so regulating mitophagy (71). In neurons, pro-mitophagy stimuli cause the translocation of the phospholipid cardiolipin from IMM to OMM, thus favoring the recognition of damaged mitochondria by the autophagic machinery in a PINK1–PARKIN-independent way (72). Intriguingly, Toyama et al. describe that energy-sensing adenosine monophosphate (AMP)-activated protein kinase (AMPK), a positive regulator of ULK1 that activates autophagy for maintaining the energy homeostasis, triggers fission activation, and consequently mitophagy, through phosphorylation of MFF, thus unveiling a new central role for AMPK in the regulation of mitochondria homeostasis (73).

Previous studies have also identified the BH3-only protein NIX (Bnip3L) and BCL2/adenovirus E1B 19 kDa protein-interacting protein 3 (BNIP3) as mitophagy receptors due to their direct interaction with LC3 (74); during the maturation of erythroid cells, NIX is involved in programmed removal of mitochondria (75), while both BNIP3 and NIX are key players in hypoxia-induced mitophagy (74, 75). In addition to mitophagy, a new pathway for mitochondrial quality control has been recently discovered (76) in which PINK1–PARKIN drive the formation of mitochondrial-derived vesicles for the delivery of mitochondrial oxidized proteins to lysosomes [reviewed by Sugiura et al. (76)].

### Mitochondria in Cancer: A Question of Balance between Energy Production and Clearance

Recent publications have identified a linear relation between mitochondria fate and cancer (77–81). In fact, mitochondria can be connected to cancer formation and progression, and their contribution has a strong impact on invasiveness and metastatic profile of cancers (79). A growing body of evidence suggests that many cancer cell lines and solid tumors undergo a drastic metabolic reprogramming; they limit the tricarboxylic acid cycle (TCA) and mitochondrial OXPHOS as a consequence of mutations that affect the activity of TCA key enzymes or the activity of OXPHOS complexes (77, 82). Therefore, cancer cells veer toward a prominent use of glycolysis as the main source for ATP production (“Warburg effect”) (83), so upregulating glucose uptake [reviewed by Gaude and Frezza (81)]. However, the connection between mitochondria dysfunction and cancer is not just related to metabolism (83). In fact, mitochondria activities can also directly or indirectly affect nuclear or mitochondrial DNA expression (84) and mutations (81), epigenetic changes (such as methylation) (77), cell migration (78), and cell death (85). Moreover, an imbalance in the mitochondrial degradation process results in progressive alteration of cellular homeostasis, which, in turn, may set the stage for the development of tumor cells. This suggests the existence of an intricate reciprocal interplay between mitochondria, autophagy/mitophagy, and tumor initiation. Autophagy has always been perceived as playing a double-faceted role in tumorigenesis, either supporting survival or promoting death, depending on the different cellular contexts. For instance, mice with heterozygotic deletion of *Becn1* are susceptible to spontaneous tumors, while deletion of other key autophagic genes such as *Atg5* or *Atg7* leads to the appearance of only benign liver tumors (86). By contrast, some types of tumors are dependent on the activation of autophagy, such as RAS-driven cancers (86, 87). In other cases, tumor cells can increase autophagy to promote chemo- and radio-resistance (88).

Intriguingly, autophagy has been described as being critical for innate and adaptive immunity through regulating antigens processing and presentation (89); moreover, autophagy induction helps the host immune system to properly recognize and eliminate pre-malignant and malignant cells (90). Recently, Pietrocola and collaborators have demonstrated that short-term fasting or autophagy-inducing caloric restriction mimetics enhances anticancer immune responses by activating immune effector T lymphocytes, thus preventing cancer cells escaping from immuno-surveillance (91). This activation of autophagy, in cancer cells, leads to their ATP release into the extracellular space where ATP acts as a chemotactic factor attracting T cells and, thus, favoring tumor growth reduction (91). Considering that mitochondria elongation correlates with more performing mitochondria and more *cristae* that should guarantee a sustained ATP production (92), we speculate that, in Pietrocola’s system mitochondrial dynamics might have a role. Thus, ideally, by pharmacologically modulating mitochondrial dynamics of tumor cells, we might act on and improve anticancer immuno-surveillance. These assumptions reinforce the belief that mitochondrial dynamics and autophagy/mitophagy are coevolutionarily interconnected along all the cell types.

In line with these notions, increasing evidence interestingly also links dysfunctions in mitophagy to cancer development (65, 76, 80, 93), even though how they are connected it is strictly dependent on the cancer type. A pathological decrease of mitophagy efficiency causes accumulation of damaged mitochondria. This leads to disruption of redox balance and to an increase of detrimental oxidative damage, such as ROS-induced DNA mutations; hence, the increase in free radicals production raises the possibility of tumors developing due to genetic instability (94, 95). Conversely, mitophagy can protect cells from apoptosis and promote tumor cell survival under some adverse conditions (71, 96).

PARKIN deletions or loss of function mutations have been identified in ovarian, breast, bladder, and lung cancers (this topic has recently been thoroughly reviewed elsewhere) (97); additionally, mice null for PARKIN develop spontaneous macroscopic hepatic tumors (98). Mutation in *PARK6* gene (PINK1) (99) has been observed in neuroblastoma, so raising the hypothesis that alteration of mitophagy may have a causal role in certain tumors. As described above, AMBRA1 is a PARKIN-interacting protein indispensable for the final step of PARKIN-triggered mitophagy (70). Ambra1 heterozygosity, in mice, has been recently associated to tumorigenesis, this gene thus acting as a tumor suppressor gene (100). Although the exact role of AMBRA1 in cancer insurgence is still largely unclear, one of the possible mechanisms, proposed by Strappazzon and Cecconi, hypothesizes that AMBRA1 dysregulation in mitophagy might be related to carcinogenesis (65). Moreover, increase in ROS levels, in particular nitric oxide levels, further impairs PARKIN E3 ligase activity (101), thus exacerbating the accumulation of damaged mitochondria, on a feedback loop. On the other hand, mitophagy is triggered by mild oxidative stress through HIF-1α (102), a transcriptional factor normally activated during hypoxic conditions, which positively regulates the expression of BNIP3 and NIX, these thus acting as tumor suppressors (93).

As already mentioned above, some tumors can use mitophagy to escape cell death activation, or as an adaptive mechanisms during the first stages of solid tumor development, when cancer cells are in the typical hypoxia environment (71, 96, 103). In fact, K-ras-induced lung tumors require mitophagy to sustain mitochondrial function and lipid catabolism and, in this case, inhibition of mitophagy leads to proliferative arrest, so negatively altering the tumor fate (104). In addition, a recent study indicates that AMPK confers metabolic stress resistance on leukemia-initiating cells and promotes leukemogenesis by suppressing ROS production and maintenance of metabolism (105), this probably also through mitophagy activation.

Interestingly, it has been demonstrated that cancer cells can act as metabolic parasites and extract nutrients from host cells by inducing catabolic processes (autophagy, mitophagy, aerobic glycolysis, and lipolysis) (106). In these cases, fibroblasts adjacent to the tumor (cancer-associated fibroblasts) positively regulate autophagy and mitophagy in order to support cancer cells metabolic needs for growth, proliferation, migration, and invasion (106–108). This demonstrates that tumor cells are able to coordinate these two pathways in different ways as a protective mechanism to ensure their own survival.

In this context, also some OMM proteins play a fundamental role in driving and promoting cancer cell progression. Recently, VDAC1, a voltage-dependent anion channel (109, 110), contributes to the metabolic phenotype of cancer cells regulating mitochondrial activity and glucose metabolism. In fact, VDAC1 directly binds to hexokinase II (HK II), so endorsing its activity (111). HK is an enzyme that catalyzes the first reaction of glycolysis, which is upregulated in broad variety of tumor types sustaining elevated rate of glucose catabolism and consequently rapid growth rates (111). Moreover, the interaction between VDAC1 and HK II inhibits mitochondrial-induced apoptosis, so helping tumor cells to elude cell death (110); furthermore, HK dissociation from mitochondria can be also a strong pro-apoptotic stimulus independent from VDAC1 (112). Though, VDAC1 is over-expressed in many cancer types, and its silencing inhibits tumor development (113). Furthermore, as described above, VDAC1 is a mitochondrial target of PARKIN, required for its efficient targeting to damaged mitochondria (62) and, therefore, essential for PINK1/PARKIN-mediated mitophagy (61). This demonstrates that, beside fusion and fission proteins, other OMM proteins have a role in the intricate interconnection between mitochondria fate and cancer.

### Mitochondrial Dynamics Are Crucial for Tumor Cell Fate Decision

Hitherto, we have discussed the role of mitophagy regulation in the progression of cancer, highlighting some link between this process and mitochondrial dynamics. But which is the actual effect of mitochondrial dynamics in tumor? Like a healthy cell, a cancer cell can undergo mitochondrial morphology changes for responding to the external environment. This decision is critical for cancer cell fate determination during tumor progression see Figure 1. In fact, cancer cells might undergo extensive fusion to promote respiratory capacity, to support proliferation, or to promote cell survival in adverse conditions, such as upon anticancer treatment (114) or glucose deprivation (115), for escaping apoptosis activation. On the other hand, cancer cells might foster fission to repress oxidative metabolism, preventing ROS formation and oxidative damage, or most likely to encourage activation of mitophagy, as mentioned. In other cases, tumor cells rely on glutamine-dependent reductive carboxylation. They upregulate mitochondrial biogenesis and increase mitochondrial respiratory chain capacity through the activation of the potent oncogene C-MYC, which, in turn, regulates the mitochondrial network controlling the expression of multiple mitochondrial genes (116, 117). From a molecular viewpoint, and related to tumor progression and severity, more than its onset, alteration of DRP1 activity seems to be a common feature of many cancer cells that, therefore, exhibit fragmented mitochondria (78, 118–123). In pancreatic cancer, DRP1 activation by ERK2 and mitochondrial fission are crucial for malignancies (119). In brain tumor initiating cells, cyclin-dependent kinase 5 (CDK5)-mediated DRP1 activation drives massive mitochondrial fragmentation, affects AMPK pathway, and correlates with poor prognosis (123). In lung cancer cells, increased mitochondrial fission is due to an upregulation of DRP1 expression and an increased ratio of Ser616-to-Ser637 phosphorylation, paralleled by a decrease of MFN2 levels, and impaired fusion. Reverting the DRP1/MFN2 imbalances result in *in vitro* reduction of cancer cell proliferation and activation of apoptosis (118). No less importantly, the coordination between mitochondrial dynamics and mitosis (so-called mitotic fission), which ensures equitable distribution of mitochondria to daughter cells, plays a role in cancer, clearly related to the requirement for mitochondrial division during mitosis. This phenomenon is regulated by cyclin B1–CDK1, which simultaneously initiates mitosis and activates DRP1 by phosphorylating its Ser616 (121). Another mitotic kinase, Aurora A, phosphorylates the Ras-like GTPase (RALA), leading to mitotic mitochondrial accumulation of RALA and its effector, ralA binding protein 1 (RALBP1). RALBP1 acts as a scaffold for recruiting DRP1 and cyclin-CDK to mitochondria and inducing fission (31).

DRP1 activation, however, is closely linked to migration and invasion of tumor cells. It has been described that imbalance of the mitochondria phenotype toward fragmentation coordinates the migratory capability in cells where migration represents a crucial physiological function, such as T lymphocytes (124). Indeed, ample data have started to highlight a strict correlation between the levels of DRP1-mediated mitochondrial fission and diverse metastatic states of different tumoral cells (125); these cells boost mitochondria fission and re-localization up in order to locally fulfill their high-energy requests for migration. This is the case of invasive breast carcinoma cells that require DRP1-dependent mitochondrial fission for re-localizing the organelles to lamellipodial regions where they need to satisfy a localized growing energy demand, so marking this process as a critical early developmental step in metastatic breast cancer (78). DRP1 overexpression is also associated with malignant oncocytic thyroid tumors, positively regulating migration of thyroid cancer cells (120). In glioblastoma cells, DRP1-induced mitochondrial fission and consequent cell migration are driven by hypoxia through HIF-1α (122). In line with these observations, the perturbation of MIRO1, which is essential for mitochondrial transport, prevents the redistribution of mitochondria to the anterior of moving epithelial cancer cells, so affecting these cells migratory abilities (126). It is important to note that DRP1 and mitochondria remodeling have a constitutive leading role in the modulation of T cell metabolic shifts, this inducing differentiation of T cells, and it is crucial for the detection and clearance of tumor cells (127) into diverse populations (128). Therefore, the connection between mitochondria fragmentation and tumor development raises questions as to whether alteration of fission–fusion processes may also negatively and specifically affect the T-cell anti-tumor surveillance, thus contributing to malignant tumor prognosis.

## Conclusion

In spite of considerable progress in understanding the implications of mitochondrial dynamics and mitophagy in controlling tumor origin and progression, and these two processes emerging cross-links, many key issues remain unresolved. Several lines of evidence indicate that it is quite unreasonable to make a general statement of how autophagy and mitophagy may influence tumor. Likewise, based on emerging findings, it appears that mitochondrial shape regulation plays a critical role in the first steps of tumor and in cancer migration/invasion ability. This breaks new ground in the fundamental understanding of possible mechanisms underlying the intimate interplay between mitochondrial homeostasis and tumors.

Although, it remains to be elucidated also how different aspects of mitochondrial metabolism and dynamics may affect the anti-tumor immune response and what their relative contributions to tumor progression or clearance are. This new concept helps to broaden our knowledge and perspectives regarding tumor cure, making it possible to identify new candidate targets for therapy.

## Author Contributions

MB and FN wrote the manuscript and prepared the figures. SC wrote the manuscript, revised and financed the work.

## Conflict of Interest Statement

The authors declare that the research was conducted in the absence of any commercial or financial relationships that could be construed as a potential conflict of interest.
